# A machine learning model to simplify recognition of patients with atrial fibrillation based on diagnostic codes in Swedish primary health care

**DOI:** 10.1186/s12911-026-03491-4

**Published:** 2026-04-17

**Authors:** Anders Norrman, Caroline Wachtler, Per Wändell, Julia Eriksson, Toralph Ruge, Boel Brynedal, Jan Hasselström, Thomas Kahan, Axel C. Carlsson

**Affiliations:** 1 Division of Family Medicine and Primary Care, Department of Neurobiology, Care Sciences and Society, Karolinska Institutet, Huddinge, Sweden; 2 Academic Primary Health Care Centre, Region Stockholm, Stockholm, Sweden; 3 Center for Primary Health Care Research, Lund University, Malmö, Sweden; 4 Division of Biostatistics, Institute of Environmental Medicine, Karolinska Institutet, Stockholm, Sweden; 5 Department of Clinical Sciences Malmö, Lund University, Lund, Sweden; 6 Department of Internal Medicine, Skåne University Hospital, Malmö, Sweden; 7 Centre for Epidemiology and Community Medicine, Region Stockholm, Stockholm, Sweden; 8 Department of Global Public Health, Karolinska Institutet, Stockholm, Sweden; 9 Division of Cardiovascular Medicine, Department of Clinical Sciences, Karolinska Institutet, Danderyd Hospital, Stockholm, Sweden

**Keywords:** Artificial intelligence, Atrial fibrillation, Gradient boosting, Normalized relative influence, Prediction

## Abstract

**Background:**

Atrial fibrillation (AF) is a major risk factor for atherothrombotic complications but is often asymptomatic and undiagnosed. This study aimed to develop a machine learning model to distinguish between individuals with low and high risk of AF, using routinely collected diagnostic data from Swedish primary health care.

**Methods:**

Cases (*n* = 42,607, aged ≥ 45 years) with diagnosed new onset AF and controls (*n* = 427,169) matched by age and sex. Machine learning models stratified for age (45–69 and ≥ 70 years) and sex were developed using stochastic gradient boosting, based on number of primary health care visits during the year before the index AF diagnosis, age, and ICD-10 codes from electronic medical records 2014–2019. Performance was evaluated by AUC, sensitivity and specificity, and key predictors ranked by normalized relative influence (NRI) and odds ratios for marginal effects.

**Results:**

The most influential predictors were the number of visits (NRI: 29.9–46.3%) and age (NRI: 6.2–15.9%), followed by risk factors for AF such as heart failure, hypertension, and cardiac arrhythmias. Model AUC ranged from 0.77 to 0.79 across subgroups. Sensitivity was 0.76–0.80, and specificity 0.58–0.66, with higher sensitivity in older groups and higher specificity in younger ones. The models correctly identified 95–98% of individuals without known AF.

**Conclusions:**

The models show good predictive ability, effectively ruling out low-risk patients while identifying known risk factors. With AUC values comparable to more complex models, our approach using only visit frequency, age, and diagnoses may support initial risk assessment in primary health care for identifying individuals at risk of AF.

**Supplementary Information:**

The online version contains supplementary material available at 10.1186/s12911-026-03491-4.

## Introduction

Atrial fibrillation (AF) affects an estimated 2–3% of the general population, with prevalence rising steeply with advancing age; however, a considerable fraction of cases remains undetected [1]. AF is a major cause of atherothrombotic stroke, and early detection is crucial, as adequate anticoagulant therapy substantially reduces the risk of stroke [1]. In Sweden, the prevalence of detected AF is estimated to be 2.9% among adults above the age of 20 [2]. However, in the STROKESTOP study, 3.0% of 75- and 76-year-olds in Sweden who participated in AF screening were found to have previously undiagnosed AF, contributing to a total AF prevalence of 12.3% in that screened population [3]. ​ In the Swedish context, population-based screening with single-lead handheld ECG has been shown to increase the previously known prevalence of AF by approximately 30%, indicating that a substantial proportion of cases remain undetected without active screening [4]. To detect AF, various strategies have been proposed, including general, targeted, and opportunistic screening, but the 2024 European Society of Cardiology (ESC) Guidelines for the management of AF recommend selective approaches and do not support universal population-based screening [1].

The use of clinical data to identify risk for AF improves the detection rates of previously unrecognized AF. Many established risk factors and risk markers are associated with incident AF, including older age, male sex, low socioeconomic status, hypertension, heart failure, coronary heart disease, prior atherothrombotic stroke, diabetes mellitus, elevated B-type natriuretic peptide (BNP), elevated C-reactive protein, low estimated glomerular filtration rate (eGFR), body mass index (BMI), and electrocardiographic (ECG) abnormalities [1]. The use of machine learning (ML) methods is becoming increasingly common in studies aiming to enhance the classification of patients at high risk of undetected chronic diseases, including AF [5], potentially offering a complement or an alternative to traditional risk prediction models [6–8]. Previous ML models for AF prediction have been developed using diverse data types, including electronic medical records, biomarkers, imaging findings, and electrophysiological parameters, reflecting the evolving landscape of AF risk stratification [9]. Many of these models, however, rely on extensive sets of clinical variables, reducing their feasibility for clinical application [10–12]. In primary health care (PHC), efficient use of limited resources and time are important creating a need to limit screening those at the highest risk of having undiagnosed AF -- those who would benefit the most from timely detection and treatment [13]. There is no population-based AF screening program in Stockholm County; however, patients undergoing evaluation for atherothrombotic stroke, acute coronary syndromes, or other cardiovascular symptoms routinely undergo ECG assessment in secondary care, and pulse palpation during blood pressure measurement is widely recommended.

We hypothesized that routinely collected register data, such as number of PHC visits, age, and International Statistical Classification of Diseases and Related Health Problems (ICD-10) codes, might predict individuals who would benefit from further evaluation, such as ECG or pulse palpation. These features are standardized, widely available, and easily extracted, which could make them suitable for scalable applications in real-world PHC settings. Thus, the aim of this study was to develop ML models based solely on routinely collected PHC data to distinguish between individuals at low and high risk of AF, supporting more efficient targeted screening strategies, which may ultimately contribute to prevent future atherothrombotic stroke.

## Methods

### Study design

This study employed a case–control design using routinely collected electronic medical records from PHC in Region Stockholm, Sweden to examine whether a limited set of standardized clinical variables can distinguish between individuals at low and high risk of AF.

### Study context

Data were collected from patients in the Stockholm Region, which had a population of approximately 2.4 million residents at the time of the study. Data for this study was gathered from the Stockholm Region regional health care data warehouse (known as VAL-databasen), where healthcare data, including diagnoses (according to ICD-10), medical visits, and hospitalization at all levels of care are systematically recorded. A dataset of comprehensive healthcare information from all levels of care within the Stockholm Region health care is available at Karolinska Institutet for research purposes (Multimorbidity Integrated Registry Across Care Levels in Stockholm, MIRACLE-S). The study adheres to the TRIPOD-AI reporting guidelines for transparent reporting of multivariable prediction models developed for individual prognosis or diagnosis [14].

### Participants

Data for this study was gathered from cases and controls were identified based on electronic medical records data from the years 2014–2019. All individuals who had received an ICD-10 diagnosis of atrial fibrillation or atrial flutter (I48) recorded in connection with any healthcare visit, in primary or secondary care, between 2010 and 2013 were excluded. Atrial fibrillation or atrial flutter (I48), identified either in primary or secondary care settings, are collectively referred to as AF in this study. In Swedish PHC, atrial fibrillation and atrial flutter are not consistently distinguished in routine documentation; therefore, reliable separation between the two conditions was not feasible in this dataset, and at the stage of risk stratification, such differentiation is not clinically meaningful. Cases were defined as individuals aged ≥ 45 years, registered at one or more PHC centers in Region Stockholm [15], who had received the ICD-10 code for atrial fibrillation or atrial flutter (I48) recorded in connection with health care visit during 2014–2019. The lower age limit of 45 years was chosen based on Swedish population data demonstrating that the prevalence of AF is very low below 45 years of age and increases markedly with advancing age thereafter [16]. Between 2014 and 2019, 43,771 individuals were identified with a newly registered diagnosis of AF. For each identified case, up to ten age- and sex-matched controls were selected from the same time period (2014–2019). In some instances, fewer than ten suitable controls were available. If more than ten eligible matched candidates were available, the ten controls were selected randomly. Sampling was performed without replacement, meaning that no individual was included more than once in the control group. After limiting the population to only include individuals ≥ 45 years of age, we ended up with a study population of 42,607 newly diagnosed patients with AF and 427,169 controls.

Whether this diagnosis was made for the first time in PHC or had been established previously elsewhere in Sweden or abroad could not be determined. The correctness of the AF diagnosis was not validated. The first time the diagnosis was recorded was defined as the index date. How a documented diagnosis was established and whether the controls underwent any investigations to screen for AF is not evident from the available data.

Age and male sex are known risk factors for AF, and the number of consultations has proven to be a strong predictor in similar ML-models for other chronic diseases [17]. Including all diagnoses above a minimum frequency has been shown to improve model performance in similar models for the detection of hypertension and diabetes, demonstrating high ability to discriminate between cases and non-cases [17, 18]. Data collected include age and sex, the number of PHC visits for 12 months preceding the index date, and all registered primary and secondary diagnoses, categorized by three-digit ICD-10 codes, recorded during all visits to a PHC centre between 2014 and 2019. First, a three-year retrospective collection of diagnoses was performed for all individuals, starting from the date of the new AF diagnosis or the corresponding index date for controls, meaning that the index date for controls could occur on a date that did not coincide with a healthcare visit generating a diagnosis. To minimize this potential bias, we included a three-year retrospective collection of diagnoses. A 3-year window was chosen as a 1-year window has been shown to catch 56% of patients with diabetes and 59% of patients with hypertension compared to a 5-year window and a 5-year window would limit the time to find AF events [19]. Diagnoses were chosen from the 2,000 most frequently registered ICD-10 codes in PHC. Of these, only diagnoses with at least 50 occurrences in the studied population were included, resulting in a total of 1,309 diagnoses [20]. This was done to exclude rare diagnostic codes. These were identified as predictors alongside number of PHC visits 12 months prior AF diagnosis or index date for controls and age.

### Variables

To further strengthen the likelihood that the cases studied represent incident AF, in addition to excluding patients with AF diagnosis between 2010 and 2013, we also excluded diagnoses indicating a potential history of post-stroke AF as predictors. Specifically, we excluded the diagnoses *a personal history of medical treatment (*Z92), including anticoagulant use (Z92.1), as well as those with *a personal history of certain other conditions* (ICD-10 Z86), such as previous TIA (Z86.6) or stroke (Z86.7c). This exclusion was based on preliminary analyses in which these variables showed disproportionately high NRI values, suggesting that they primarily captured prior disease history rather than incident risk, and were therefore removed to improve transparency and interpretability of the final model.

Several diagnoses were merged and treated as single diagnostic predictor categories in the model. A complete list of these merged categories, including diabetes (E10–E13), hypertension (I10–I15), and coronary heart disease (I20, I21, I23, I24, I25) is provided in Supplementary Table 1. Type 1 and type 2 diabetes were analyzed together, as many individuals had multiple ICD-10 codes for diabetes, making separation based solely on diagnostic coding unreliable [19].

### Statistical analyses

Data were analyzed using stochastic gradient boosting (SGB), a ML method that has previously been shown to be effective in predictive modelling of other chronic conditions within PHC settings [21, 22].

We developed four separate models to account for the substantial differences in search patterns, diagnostic characteristics, and incidence rates of AF observed across age and sex [20]. To account for differences in AF prevalence and the distribution of risk factors, the study population was stratified by sex and age group: women and men aged 45–69 years and ≥ 70 years.

### Machine learning models

The four SGB models used a Bernoulli loss function fitted to 20 000 trees, each having a maximum depth of 5 interactions, with a shrinkage (learning rate) of 0.001, a minimum of 10 observations in the terminal nodes of the trees, and a subsampling rate (bag fraction) of 0.5. The optimal number of trees to use for prediction was estimated using 10-fold cross validation. The optimal number of trees was used on the test data to predict AF.

### Model validation

Within each SGB model, the data was further divided into a 50% training set and a 50% test set, to ensure robust model development and evaluation. The performances of the four models were assessed using ROC curves, sensitivity, and specificity. The SGB models were then applied to each test data set to obtain patient specific probabilities of being newly diagnosed with AF. The probabilities that maximized the sum of sensitivity and specificity were used as a cut-off value, such that patients with a probability higher than this cut-off were classified as being newly diagnosed with AF.

Using the SGB models, we identified and ranked the predictors most strongly associated with newly diagnosed AF, represented by their normalized relative influence (NRI) scores and corresponding odds ratios of marginal effects (OR_ME_). For each predictor, the odds ratio was calculated using the probabilities of being newly diagnosed with AF obtained by integrating out all other variables in the model using the weighted tree traversal method.

All four models were assessments of their (ROC) curve performance and evaluated the NRI of predictors, including registered ICD-codes in PHC, age, and the number of PHC visits 12 months preceding the index date. For each of the four training datasets we included age, number of PHC visits 12 months prior and diagnoses with at least 10 occurrences. This resulted in 316 diagnoses for women aged 45–69, 449 for women aged ≥ 70, 427 for men aged 45–69, and 468 for men aged ≥ 70.

The analyses were performed using R version 4.2.1 [23].

## Results

### General

A total of 42,607 cases, 15,298 individuals aged 45–69 years and 27,309 aged 70 years and older, were diagnosed with AF. A summary of included cases and controls according to sex and age group is presented in Table 1.

**Table 1:** Participant characteristics for the cases and controls included in the overall study population, and in the training and test datasets

Variable	Total		Training dataset		Test dataset	
	Cases	Controls	Cases	Controls	Cases	Controls
All participants	42,607	427,169	21,528	213,362	21,079	213,807
Age at index date, mean (SD)^a^	73.0 (9.3)	69.9 (9.4)				
Women 45–69 years, n (%)	5,189	76,216	2,623	38,080	2,566	38,136
Women ≥ 70 years, n (%)	13,986	118,425	7,050	59,156	6,936	59,269
Men 45–69 years, n (%)	10,109	146,292	5,104	73,097	5,005	73,195
Men ≥ 70 years, n (%)	13,323	86,236	6,751	43,029	6,572	43,207

### Model performance

The sensitivities, specificites, and AUC for predicting new AF in the test set by sex and age group are shown in Table 2. The sensitivity ranged between 0.76 and 0.80 (highest in the ≥ 70 year age groups for both women and men), and specificity ranged between 0.58 and 0.66 (highest in the 45–69 years age groups). Overall, the four models demonstrated similar AUCs across age and sex groups, ranging from 0.77 to 0.79.

**Table 2:** Statistical performance of the models in the test data sets

Group	Sensitivity	Specificity	AUC
Women 45–69 years	0.78	0.64	0.785 (0.776–0.793)
Women ≥ 70 years	0.80	0.58	0.765 (0.760–0.771)
Men 45–69 years	0.77	0.66	0.788 (0.783–0.794)
Men ≥ 70 years	0.83	0.71	0.778 (0.772–0.783)

Estimates for sensitivity and specificity for the age- and sex‐stratified machine learning models, with area under curve (AUC) and 95% confidence intervals (CI)

The confusion matrices for women and men by age group, together with the corresponding positive and negative predictive values, are presented in Table 3. The models demonstrated 95–98% ability to correctly classify patients in all groups without a recorded diagnosis of AF. Positive predictive values ranged from 14% to 37% in the models, with the highest fraction among men ≥ 70 years. All four SGB models reached the optimal number of trees, ranging between 1900 and 3764 (see Supplementary Table 2).

**Table 3:** Confusion matrix for predicting presence of incident atrial fibrillation using the optimal stochastic gradient in the test data set

Predicted	Observed		
	No atrial fibrillation	Atrial fibrillation	Total
**Women 45–69 years**			
No atrial fibrillation n (%)	24,205 (97.8)	553 (2.2)	24,758
Atrial fibrillation n (%)	13,931 (87.4)	2,013 (12.6)	15,944
Total n (%)	38,136 (93.7)	2,566 (6.3)	40,702
Positive predictive value		0.13	
Negative predictive value	0.98		
**Women ≥ 70 years**			
No atrial fibrillation n (%)	34,374 (96.2)	1,363 (3.8)	35,737
Atrial fibrillation n (%)	24,895 (81.7)	5,573 (18.3)	30,468
Total n (%)	59,269 (89.5)	6,936 (10.5)	66,205
Positive predictive value		0.18	
Negative predictive value	0.96		
**Men 45–69 years**			
No atrial fibrillation n (%)	48,315 (97.6)	1,176 (2.4)	49,491
Atrial fibrillation n (%)	24,880 (86.7)	3,829 (13.3)	28,709
Total n (%)	73,195 (93.6)	5,005 (6.4)	78,200
Positive predictive value		0.13	
Negative predictive value	0.98		
**Men ≥ 70 years n (%)**			
No atrial fibrillation n (%)	26,846 (95.1)	1,398 (4.9)	28,244
Atrial fibrillation n (%)	16,361 (76.0)	5,174 (14.0)	21,535
Total n (%)	43,207 (86.8)	6,572 (13.2)	49,779
Positive predictive value		0.14	
Negative predictive value	0.95		

### Variable importance

The number of PHC visits in the 12 months preceding an AF diagnosis emerged as the single strongest predictor of AF, with high effects among older individuals (e.g., OR 8.0 for men ≥ 70 years). Age at diagnosis, together with recorded diagnoses of cardiovascular conditions such as cardiac failure (I50), hypertension (I10–I15), abnormalities of heart beat (R00), and other arrhythmias (I49), showed consistently high NRI in the models, and were all strongly associated with OR_ME_ values above 1. Overall, age contributed an NRI between 5% and 16% across the four models. Tables 4 and 5 present the ten predictors with the highest NRI, along with their corresponding OR_ME_ values, by sex and stratified by age group. Encountering health services (Z76) was among the 10 most influential predictors in all four models and had an OR_ME_ slightly below 1 in all models.

**Table 4:** The top 10 predictors, including diagnoses defined by ICD-10 codes recorded in the electronic medical record, with highest normalized relative influence for predicting incident atrial fibrillation among women, along with the odds ratios for the marginal effects

Predictors	Relative influence (%)	OR_ME_
**Women 45–69 years**		
Number of visits 12 months before diagnosis	29.9	3.4
Age at diagnosis	15.9	0.4
Abnormalities of heart beat (R00)	5.9	3.0
Cardiac failure (I50)	4.6	3.5
Other cardiac arrhythmias (I49)	3.0	2.8
Presence of cardiac and vascular implants and grafts (Z95)	2.0	2.5
Hypertension (I10-I15)	1.48	1.2
Overweight and obesity (E66)	1.14	1.5
Transplanted organ and tissue status (Z94)	1.1	6.2
Encountering health services (Z76)	1.1	0.9
**Women ≥ 70 years**		
Number of visits 12 months before diagnosis	37.3	3.5
Age at diagnosis	11.9	1.6
Cardiac failure (I50)	5.1	1.7
Hypertension (I10-I15)	2.6	1.3
Abnormalities of heart beat (R00)	2.5	2.2
Other cardiac arrhythmias (I49)	2.3	3.0
Convalecence (Z54)	1.5	1.6
Encountering health services (Z76)	1.0	0.9
Observation (Z03)	0.99	1.0
Paroxysmal tachycardia (I47)	0.94	3.6

The optimal stochastic gradient boosting (SGB) model with 1,900 optimal trees for women 45–69 years and 3,148 trees for women ≥ 70. Z76 denotes: Persons encountering health services in other circumstances, Z03 Encounter for medical observation for suspected diseases and conditions ruled out, NRI normalized relative influence, OR_ME_ marginal effects

**Table 5:** The top 10 predictors, including diagnoses defined by ICD-10 codes recorded in the electronic medical record, with highest normalized relative influence for predicting incident atrial fibrillation among men, along with the odds ratios for the marginal effects

Predictors	Relative influence (%)	OR_ME_
**Men 45–69 years**		
Number of visits 12 months before diagnosis	33.7	5.5
Age at diagnosis	12.4	0.97
Cardiac failure (I50)	5.2	2.3
Abnormalities of heart beat (R00)	3.1	2.6
Other cardiac arrhythmias (I49)	2.7	4.0
Presence of cardiac and vascular implants and grafts (Z95)	1.97	1.6
Encountering health services (Z76)	1.27	0.9
Hypertension (I10-I15)	1.19	1.0
Paroxysmal tachycardia (I47)	0.94	4.6
Observation (Z03)	0.85	0.9
**Men ≥ 70 years**		
Number of visits 12 months before diagnosis	46.3	8.0
Age at diagnosis	6.2	1.34
Cardiac failure (I50)	3.9	1.7
Presence of cardiac and vascular implants and grafts (Z95)	1.4	1.6
Hypertension (I10-I15)	1.3	1.2
Other cardiac arrhythmias (I49)	1.18	2.0
Convalecence (Z54)	0.87	1.4
Abnormalities of breathing (R06)	0.83	1.3
Encountering health services (Z76)	0.82	0.9
Observation (Z03)	0.76	1.0

The optimal stochastic gradient boosting (SGB) model with 3,764 optimal trees for men 45–69 years and 3,306 trees for men ≥ 70. Z76 denotes Persons encountering health services in other circumstances, Z03 Encounter for medical observation for suspected diseases and conditions ruled out, NRI normalized relative influence, OR_ME_ marginal effects

## Discussion

### Main findings

This study demonstrates that newly diagnosed AF can be predicted using SGB models incorporating the number of PHC visits, age, and diagnostic codes from PHC during the three years preceding diagnosis. The models showed overall good discrimination (AUC 0.77–0.79), high negative predictive values (0.95–0.98), and a clinically valuable positive predictive values (0.13–0.18), with performance comparable to previously published machine learning models [16–18]. The high negative predictive values and the lower positive predictive values indicate that the model is suited as a risk strafification tool, enabling more efficient use of PHC resources while still identifying patients with AF.

Our study confirms previous evidence that AF can be predicted for risk stratification from electronic medical records, and extends these findings by demonstrating feasibility using Swedish PHC data, although with somewhat lower AUC values (0.77–0.79) than those reported in previous studies. A study evaluating different predictive models for AF prediction using harmonized electronic health record data, demonstrated that a single-layer neural network with random oversampling performed best, achieving an AUC of 0.80, solely based on diagnoses with a known association to incident AF, along with age and sex [12]. Previous studies have explored different ML models to identify undiagnosed AF using routinely collected primary care data, reporting AUC of 0.83 in a UK study [10], and AUC of 0.83 in a study based on clinical practice data in a German study [11]. Among the evaluated models, the model for men aged ≥ 70 years demonstrated the highest sensitivity (0.83) and specificity (0.71). A possible explanation is the higher prevalence of cardiovascular disease and the lower burden of competing non-cardiovascular diagnoses in this group compared with younger men and women in both age groups [20], which may contribute to improved diagnostic discrimination.

Our study suggests that risk stratification may be achievable using the models. In the current study, as shown previously [10–12], findings suggest that age and sex, together with a limited set of key diagnoses, particularly hypertension, heart failure, and cardiac arrhythmias, are sufficient to identify individuals with risk of AF as well as individuals at low risk of AF. The variable with the highest NRI was Number of visits 12 months before diagnosis and this is likely explained by an increase in the likelihood of detection of AF at every visit. The diagnoses contributing most to the prediction models were conditions with well-established association with incident AF, such as cardiac arrhythmias [11], hypertension [6, 7], and heart failure [6, 7]. Abnormalities in heartbeat (R00) was a predictor of AF and based on our understanding of Swedish PHC and the present findings, patients with R00 should be screened for AF. R00 is sometimes coded during a remote consultation and should be followed up with an in-person visit to rule out AF and other underlying conditions.

We observed that advancing age was associated with an increased risk of AF in both women and men aged ≥ 70 years, consistent with previous studies [6–8, 24]. However, in contrast to this, the models for women and men aged 45–69 years demonstrated an inverse association between increasing age and AF risk. We believe that this is an effect of the matching procedure, where individuals were matched for age. Men showed a higher incidence of AF compared to women [25]. We were not able to account for ethnicity [26] and socioeconomic status [27], both recognized as important determinants of AF risk, due to a lack of available data.

This study was unable to duplicate previous strong associations between AF and several chronic diseases, but this could be due to the nature of the data. Chronic kidney disease, hyperlipidemia, diabetes mellitus, peripheral artery disease, and coronary artery disease, also contributed to the models but did not rank among the top ten strongest predictors of AF risk. This likely reflects the underuse or underreporting of several chronic diagnoses in PHC and suggest that a five-year time window is sometimes needed to capture a larger proportion of chronic conditions [28]. Obesity [6, 7] was identified as a contributing factor but not a diagnosis of alcohol dependence; however, other lifestyle and behavioral variables including the level of alcohol consumption, smoking, physical inactivity, and family history of AF were absent from our dataset.

There are many potential explanations for why the number of PHC visits was the most contributing factor to prediction in all of the models. This aligns with previous findings in the prediction of type 2 diabetes [17], likely reflecting a higher burden of chronic comorbidities associated with risk of incident AF. Frequent healthcare use is associated with multiple chronic conditions, medically unexplained symptoms, and mental health issues such as anxiety and depression [29–31]. These patients also have more frequent opportunities to have AF recognized through clinical assessments. Persons encountering health services in other circumstances (Z76), which often represents non–face-to-face contacts such as prescription renewals, showed an OR_ME_ below 1 across all models. Although this indicates that the model may be incomplete as a pure disease-risk predictor, the finding is of interest, as it highlights that patients with frequent non–face-to-face contacts but few in-person visits may represent a group where additional opportunistic screening could be warranted. The high influence of the number of PHC visits likely reflects its status as a continuous variable, which provides more information than dichotomous diagnoses and allows the SGB models to identify both linear and nonlinear patterns.

### Strengths and limitations

The findings of this study should be interpreted in the context of its quality as an AI-based prediction model, taking into account five key criteria: transparent reporting and reproducibility, a clearly defined intended use, rigorous internal and external validation, an adequate sample size, and openness of data and software [32]. This study is reported according to the TRIPOD-AI guidelines [14], which provide updated standards for transparent reporting of prediction models developed with regression or ML methods. The reproducibility of our results is supported by our findings confirming results from similar ML models [10–12].

The study has several strengths. First, the development and validation of the model on a large, representative dataset.

Secondly, a strength of the study is that it provides a well-described and detailed account of the model, including how predictors were selected, which ML algorithms were used, and the assumptions under which they were applied. However, the study was based solely on data from Stockholm County, and external validation in independent populations and health care systems are required.

Third, an adequate sample size of this study was ensured by including all patients in Stockholm County aged 45 years and older with a new diagnosis of AF. This represents nearly one quarter of the Swedish population and resulted in more than 40,000 cases.

Forth, the model was specifically designed to predict incident rather than post-stroke AF, achieved by excluding patients with recorded diagnoses of prior TIA/stroke (Z86) or anticoagulant treatment (Z92). However, patients prescribed oral anticoagulants were not excluded, even though oral anticoagulant use might better capture prior TIA/stroke, which is not always coded. Similarly, patients with sequelae of cerebrovascular disease (I69) were not excluded; however, this variable showed low influence in the model.

Fifth, apart from regression methods, where colinearity of similar variables may be limiting, in tree-based ML models collinearity is not an issue.

The study has several limitations. First, the matched case–control design may lead to overestimation of the true prevalence and positive predictive value, as a substantial proportion of the underlying population was excluded and the analyses were conducted in a selected population with a higher prevalence than that of the general population within the studied age groups.

Second, a major limitation concerns how previously diagnosed AF was defined. We excluded all patients with a recorded AF diagnosis between 2010 and 2013. However, there is a risk that some individuals may have received an AF diagnosis in another healthcare system or before 2010, which represents a potential source of misclassification.

Third, case identification relied on diagnostic codes without information about how AF was detected or whether diagnoses were confirmed by ECG. Controls were not systematically screened, and it is likely that undiagnosed AF was present in this group. This could lead to misclassification and reduced model performance.

Fourth, no systematic screening was performed. Consequently, we cannot determine whether the model predicts the future development of AF itself or merely the likelihood of receiving a clinical diagnosis of AF.

Fifth, the study was based solely on data from Stockholm County, and the lack of external validation represents another major limitation; therefore, validation in independent populations and healthcare systems is essential before the model can be considered for clinical implementation.

Sixth, relates to the retrospective feature window starting from the date of the new AF diagnosis (and the corresponding index date for controls). As diagnoses recorded during the same encounter as the AF diagnosis were included, some predictors may reflect the diagnostic workup leading to AF detection rather than predictive features. Consequently, part of the model performance may be driven by signals related to the diagnostic encounter itself, potentially leading to an overestimation of predictive performance.

Seventh, among women and men, aged 45–69 years, the observed odds ratio for age in both groups were below 1.0 suggested an inverse association with increasing age. This counterintuitive finding is unlikely to represent a true protective effect of age but rather reflects the matching design and resulting within-stratum comparisons. Consequently, the estimated effect of age within this subgroup should be interpreted with caution.

Eight, the dominance of visit frequency may influence the interpretability of the models, as it can overshadow other predictors and reflect healthcare utilization patterns rather than disease-specific risk.

Ninth. the model does not incorporate well-established clinical risk markers for AF, which may constrain predictive accuracy. Several established factors associated with incident AF, including blood pressure values, BMI, ethnicity, tobacco use, and socioeconomic status were not available. Furthermore, diagnostic codes in our study were assigned by physicians at their discretion and are therefore subject to an inherent risk of incomplete or inaccurate reporting; a limitation that has been shown to result in underreporting of conditions such as obesity, hyperlipidemia, chronic kidney disease, and peripheral artery disease in Swedish PHC [17].

Tenth, the sample sizes of the age- and sex-stratified test datasets differed, which may have resulted in minor variation in the precision of performance estimates between strata; however, all subgroups were large and demonstrated narrow confidence intervals for AUC, indicating stable model performance.

### Implications and further directions

Findings from this study indicate that a limited set of routinely available variables in electronic medical records, age, a few key diagnoses, and the number of PHC visits, allow reliable identification of individuals unlikely to have AF. Such easily obtainable factors may thus help identify individuals who would benefit from targeted AF screening. Furthermore, such information could be integrated into automated decision-support tools within electronic medical record systems. The ML models could serve as a decision-support tool to identify patients who should undergo pulse palpation or ECG. This ML models could be accessible to all PHC staff, for example during routine visits such as suture removal, increasing task shifting and optimize work flows in PHC. Comparisons with established AF prediction scores, such as C2HEST, would be of considerable interest.

However, further studies are needed to determine whether screening strategies based on such tools can improve clinical outcomes, such as reducing the risk of stroke, as suggested, but not confirmed, by recent trials [33].

## Conclusions

The study demonstrates that ML models to predict incident AF in a PHC setting based on the number of visits during the 12 months preceding diagnosis, age, and diagnostic codes from PHC provide a high negative predictive value, indicating potential utility for risk stratification to guide targeted AF screening. However, their clinical applicability remains uncertain, as cost-effectiveness, integration into clinical workflows, and their impact on preventing atherothrombotic stroke have not been evaluated. In addition, the validity of the SGB prediction models is limited by uncertainty about whether the diagnostic data truly reflect underlying AF status and therefore warrants further study.

## Supplementary Information

Below is the link to the electronic supplementary material.

Supplementary Material 1

Supplementary Material 2

## Data Availability

The data from this study can be accessed for research by qualified researchers who have been trained in confidentiality protocols for human subjects, following ethical approval from Region Stockholm at halsodata.rst@regionstockholm.se. Analytical code and programs can be obtained from (mailto: axel.carlsson@ki.se).

## Abbreviations

AF: Atrial fibrillation
AI: Artificial intelligence
AUC: Area under the receiver operating characteristic curve
BMI: Body mass index
BNP: B–type natriuretic peptide
CRP: C–reactive protein
ECG: Electrocardiogram
ESC: European Society of Cardiology
eGFR: Estimated glomerular filtration rate
ICD: 10–International Statistical Classification of Diseases and Related Health Problems, 10th Revision
ML: Machine learning
NRI: Normalized relative influence
ORME: Odds ratio of marginal effects
PHC: Primary health care
ROC: Receiver operating characteristic
SGB: Stochastic gradient boosting
SD: Standard deviation
TIA: Transient ischemic attack
TRIPOD: AI–Transparent Reporting of a multivariable prediction model for Individual
