# Supplementary material for: A machine learning model to simplify recognition of patients with atrial fibrillation based on diagnostic codes in Swedish primary health care
Source: BMC Med Inform Decis Mak. 2026 Apr 17;26:126. doi: 10.1186/s12911-026-03491-4 (PMC13097664; doi:10.1186/s12911-026-03491-4)
Supplement: Supplementary file 1 — Supplementary Material 1 [file 12911_2026_3491_MOESM1_ESM.docx]

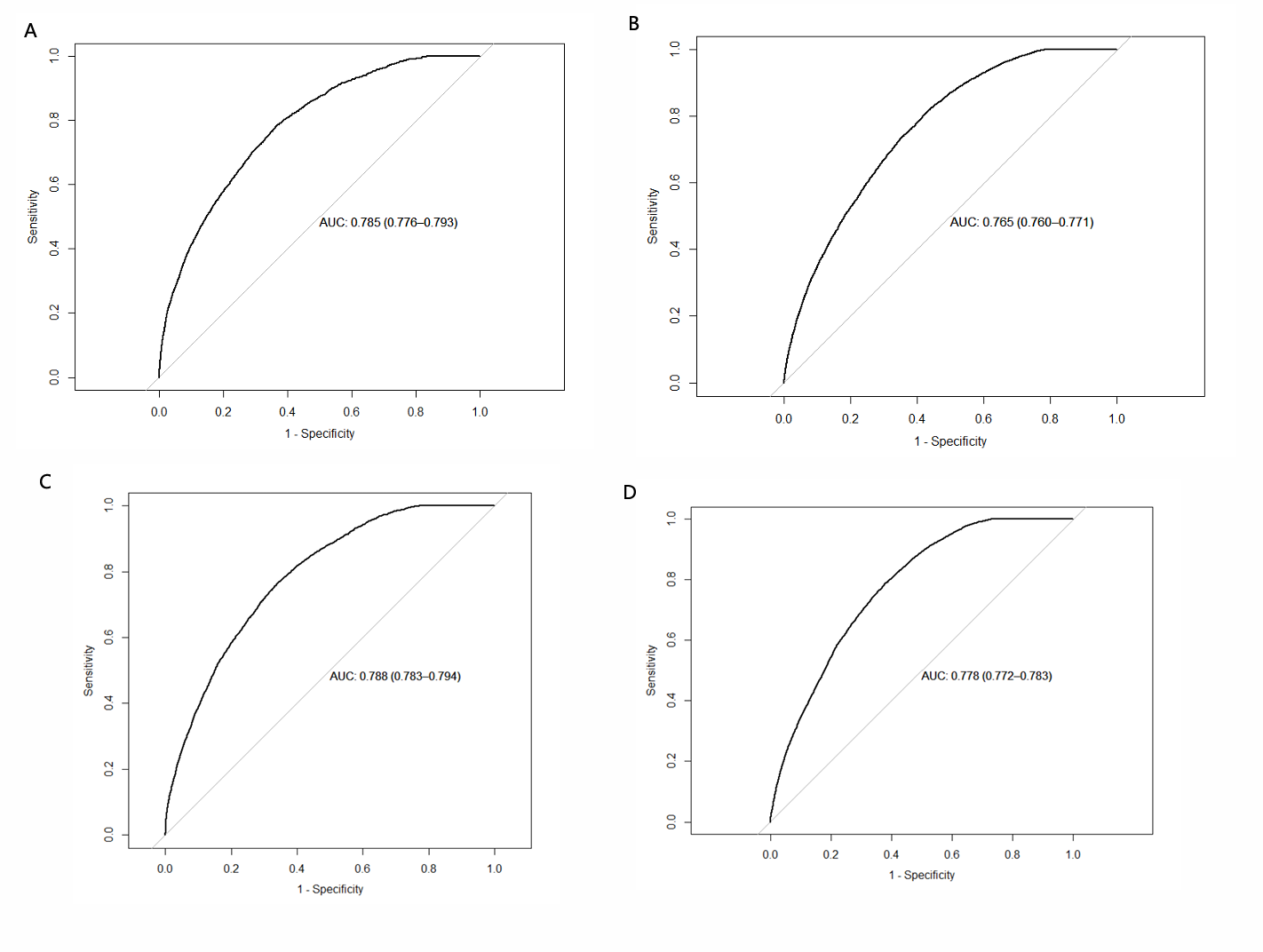


**Supplementary figure 1.** Receiver operator characteristics curve for the optimal stochastic gradient boosting model in the test data sets. Panels A–D represent women aged 45–69 years, women aged ≥70 years, men aged 45–69 years, and men aged ≥70 years, respectively.
