# Supplementary material for: A machine learning model to simplify recognition of patients with atrial fibrillation based on diagnostic codes in Swedish primary health care
Source: BMC Med Inform Decis Mak. 2026 Apr 17;26:126. doi: 10.1186/s12911-026-03491-4 (PMC13097664; doi:10.1186/s12911-026-03491-4)
Supplement: Supplementary file 2 — Supplementary Material 2 [file 12911_2026_3491_MOESM2_ESM.docx]

**Supplementary table 1.** Diagnoses that were merged into one variable containing at least two ICD-10 codes. All other ICD-10 codes were used as individual features with one letter and two digits.

| **Name** | **ICD** | **Condition** |
| --- | --- | --- |
| Asthma (current) | J45  J46 | Asthma  Acute asthma |
| Arterial conditions | I77  I79 | Arterial conditions |
| Arthritis | M50  M51  M52 | Arthritis |
| Cancer (new diagnosis in last five years) | C01-C97 | Malign tumors |
| Chronic liver disease | K72  K74  K75  K76    K77 | Chronic liver failure (K72.1)  Unspecified liver failure (K72.9)  Cirrhosis  Other specified liver disease  Unspecified liver disease  Secondary liver disease |
| Chronic obstructive pulmonary disease | J41  J42  J43  J44 | Specified chronic bronchitis  Unspecified chronic bronchitis  Emphysema  COPD |
| Chronic renal disease | N18  N19  I12   N05  N25 | Chronic renal disease  Unspecified chronic renal disease  Hypertensive renal disease w/ renal failure  Nephritic syndromes  Tubular renal disease |
| Diabetes | E10  E11  E12  E13  E14 | DM type 1  DM type 2  Starvation related DM  Other specified DM  Unspecified DM |
| Dyspepsia (current) | K29          K30 | Other specified gastritis (K29.6)  Chronic unspecified gastritis (K29.4)  Chronic atrophic gastritis (K29.5)  Chronic superficial gastritis (K 29.3)  Unspecified gastritis (K29.7)  Dyspepsia |
| Encephalitis | G04  G05  B86  B94 | Encephalitis    Post encephalitis |
| Glaucoma | H40  H42 | Primary glaucoma  Secondary glaucoma |
| Hearing loss | H91  H92 | Specified hearing loss  General hearing loss |
| Hypertension | I10  I11  I12  I13  I15 | Essential hypertension,  Hypertensive heart disease, (I119)  Hypertensive kidney disease,  Hypertensive heart and kidney disease  Secondary hypertension |
| Inflammatory bowel disease | K50  K51 | Crohn’s disease  Ulcerous colitis |
| Myokarditis | I40  I41 | Myocardia |
| Prostate disorders | N40  N41  N42 | Prostate enlargement  Prostatitis  Other prostate disease |
| Eczema | L20  L23  L24  L25  L26  L30 | Allergic eczema  Allergic contact eczema  Irritative contact eczema  Unspecified contact eczema  Exfoliative dermatitis  Eczema |
| Rheumatoid arthritis, other inflammatory polyarthropathies & systematic connective tissue disorders | M05  M06  M07  M08  M09  M11  M12  M30  M31  M32  M33  M34  M35 | Seropositive rheumatoid arthritis  Rheumatoid arthritis  Psoriasis arthropathy  Juvenile rheumatoid arthritis  Juvenile arthritis + other disease  Pyrophosphat arthritis  Other sinovitis  Specific polyarthritis  Specific systemic tissue disorders  SLE  Myositis  Sclerosis  Other systemic tissue disorders |
|  |  |  |
| Stroke & transient ischaemic attack | I60  I61  I62  I63  I64  I65    I66    I67  I68  I69  I74  I80 | Subarachnoidal hemorrhage  Intracerebral hemorrhage  Subdural hemorrhage  Cerebral infarcation  Acute cerebrovascular disease  Occlusion and stenosis of precerebral arteries  Occlusion and stenosis of intracraniell arteries  Aneurysm  Other cerebrovascular disease  Late effects of cerebrovascular disease  Cerebral infarction |
| Hyperthyroid disorders | E01  E04  E05  E06 | Iodine related struma  Atoxic struma  Thyroideotoxicosis  Thyroiditis |
| Hypothyroid disorders | E02  E03  E07 | Subclinical hypothyroid  Hypothyroidism  Other thyroid disease |
| Venous thromboembolism | I26  I27  I80  I82 | Blood clots in veins  Lung embolism |
| Viral Hepatitis | B16  B17  B18  B19 | Hepatitus B  Acute hepatitus C, D, E  Chronic hepatitus  Unspecified viral hepatitis |

**Supplementary table 2.** Optimal number of trees in the four stochastic gradient boosting models.

|  | Optimal number of trees |
| --- | --- |
| Women 45–69 years old | 1900 |
| Women ≥70 years old | 3148 |
| Men 45–69 years old | 3764 |
| Men ≥70 years old | 3306 |
